# Engineered bacteria and engineered probiotics for intestinal disease therapy: a bibliometric and knowledge mapping analysis

**DOI:** 10.3389/fmicb.2026.1970221

**Published:** 2026-09-24

**Authors:** Wei Sun, Hongru Liu, Anqi Shao, Rui Li, Wenyue Ma, Mingyu Zheng, Shengying Yan, Shuhan Liu, Minjie Wu, Daqing Sun

**Affiliations:** 1 Department of Pediatric Surgery, Tianjin Medical University General Hospital, Tianjin, China; 2 Department of Neurosurgery, The First Medical Center of the Chinese PLA General Hospital, Beijing, China

**Keywords:** bibliometric analysis, colorectal cancer, engineered bacteria, engineered probiotics, inflammatory bowel disease, intestinal diseases, knowledge mapping, synthetic biology

## Abstract

**Objective:**

Engineered bacteria and engineered probiotics are emerging living therapeutic platforms for intestinal diseases, supported by advances in synthetic biology, genetic engineering, and microbiome technologies. This study aimed to characterize the research landscape, knowledge structure, research hotspots, and emerging trends of engineered bacteria and engineered probiotics for intestinal disease therapy using bibliometric and knowledge mapping approaches.

**Methods:**

Publications were retrieved from the Web of Science Core Collection and Scopus databases. After publication type restriction, thematic relevance screening, database merging, and duplicate removal, 499 publications published between 2015 and 2026 were included. The search was completed on July 3, 2026; therefore, records from 2026 represent a partial-year dataset. R, Python, VOSviewer, and CiteSpace were used to analyze annual publication trends, country/region, institution, author and journal distributions, keyword co-occurrence and bursts, co-cited references, and research frontiers.

**Results:**

Publication output increased markedly over the study period, reaching 109 publications in 2025; 64 records were retrieved for 2026 by the cutoff date. China had the highest publication output, whereas the United States had the highest total link strength, and Asia contributed 361 publications. Keyword analysis identified inflammatory bowel disease, gut microbiota, probiotics, engineered bacteria, engineered probiotics, synthetic biology, intestinal barrier, drug delivery, colorectal cancer, and immunotherapy as major research hotspots. Co-cited reference and timeline analyses indicated a shift in research attention from conventional probiotic-mediated anti-inflammatory effects, mucosal immune regulation, and intestinal barrier protection toward bacterial chassis selection, enhanced colonization, self-regulated genetic circuits, probiotic-based biomaterials, sustained-release delivery systems, and engineered bacteria-mediated antitumor therapy.

**Conclusion:**

The bibliometric evidence indicates that recent research attention is increasingly focused on programmable living therapeutic strategies. Environmental sensing, local therapeutic delivery, sustained activity, and multifunctional design features are capabilities reported in the underlying literature and represented among the emerging themes identified in this analysis, rather than therapeutic performance directly established by the bibliometric data. Future research should further address biosafety, genetic stability, spatiotemporal controllability, intestinal colonization, delivery efficiency, and clinical translation.

## 1 Introduction

Intestinal diseases represent a major spectrum of disorders that substantially affect human health worldwide, including inflammatory bowel disease (IBD), colorectal cancer (CRC), intestinal infections, radiation-induced intestinal injury, and functional bowel disorders. IBD and CRC are two representative examples of this growing disease burden. The global prevalence of IBD has continued to increase, with approximately 6.8 million affected individuals reported worldwide in 2017 (Ng et al., 2017; GBD 2017 Inflammatory Bowel Disease Collaborators, 2019). CRC is also one of the most common malignancies globally, with more than 1.9 million new cases and approximately 0.9 million deaths reported in 2022 (Bray et al., 2024). The initiation and progression of these intestinal diseases are commonly associated with gut microbial dysbiosis, disruption of the mucosal barrier, impaired immune homeostasis, and sustained inflammatory responses. In particular, IBD is closely linked to the reciprocal imbalance among the gut microbiota, epithelial barrier, and immune system, whereas CRC development is influenced by microbial dysbiosis, barrier dysfunction, chronic inflammation, and remodeling of the tumor immune microenvironment (Vindigni et al., 2016; Chen et al., 2017; Wirbel et al., 2019; Genua et al., 2021; Shan et al., 2021).

Current therapeutic strategies for intestinal diseases include anti-inflammatory agents, immunosuppressants, biologics, antibiotics, fecal microbiota transplantation, and surgical intervention, as well as radiotherapy, chemotherapy, molecular targeted therapy, and immunotherapy for CRC. These approaches have improved clinical outcomes to some extent. However, several limitations remain in clinical practice, including marked interindividual variability in therapeutic response, safety concerns associated with long-term treatment, high recurrence rates, increasing drug resistance, limited targeting capacity, high treatment costs, and an insufficient ability to dynamically regulate the complex intestinal microenvironment (Riglar and Silver, 2018; Wirbel et al., 2019). Therefore, the development of more precise and safer therapeutic strategies that can exert sustained local effects within the intestine remains an important scientific challenge and clinical need.

In recent years, advances in synthetic biology, genetic engineering, and microbiome technologies have promoted the development of engineered bacteria and engineered probiotics as emerging therapeutic platforms for intestinal diseases. These platforms have attracted increasing attention because the underlying literature reports capabilities including intestinal colonization, environmental sensing, *in situ* production of therapeutic molecules, and modulation of microbial or immune homeostasis (Ozdemir et al., 2018; Riglar and Silver, 2018; Charbonneau et al., 2020; Rutter et al., 2022). Through genetic circuit design and chassis engineering, engineered bacteria can also be programmed to respond to disease-associated signals such as inflammation, oxidative stress, hypoxia, specific metabolites, or pathogen-associated signals (Cubillos-Ruiz et al., 2021).

Reported applications in the underlying literature include local delivery of anti-inflammatory factors and other therapeutic payloads, modulation of gut microbial communities, bacteria-mediated tumor therapy, and integration with biomaterials, microcapsules, nanoscale delivery systems, or outer membrane vesicles (Isabella et al., 2018; Wu et al., 2024; Guo et al., 2024; Carolak et al., 2026; Din et al., 2016; Gurbatri et al., 2020; Cheng et al., 2021; Ma et al., 2023; Cook et al., 2012; Dodoo et al., 2017). These diverse directions highlight the interdisciplinary nature of the field and the need for a systematic quantitative overview of how its research themes and knowledge structure have developed.

To date, existing bibliometric studies have mainly focused on relatively specific topics, including the gut microbiota in IBD, the gut microbiota in CRC, the gut microbiota in intestinal diseases, and bacteria-mediated cancer therapy; a recent study also examined genetically engineered bacteria in tumor therapy (Wu et al., 2023; Zhang et al., 2023; Zhu Y. et al., 2026). However, a cross-disease bibliometric analysis centered specifically on engineered bacteria and engineered probiotics for intestinal disease therapy remains limited. Given the rapid growth and interdisciplinary convergence of synthetic biology, living drug delivery, gut microbial regulation, and tumor immunotherapy, it is important to characterize publication development, collaboration patterns, thematic structure, research hotspots, knowledge bases, and emerging trends. Therefore, based on the Web of Science Core Collection and Scopus databases, this study performed a bibliometric and visualization analysis to systematically characterize the research landscape, knowledge base, research hotspots and emerging trends of engineered bacteria and engineered probiotics for intestinal disease therapy. The findings are expected to provide a quantitative reference for future basic research, translational studies, and clinical applications in this rapidly developing field.

## 2 Materials and methods

### 2.1 Data acquisition and search strategy

The data for this study were retrieved from the Web of Science Core Collection (WoSCC) and Scopus databases. WoSCC is a widely used multidisciplinary citation database in bibliometric research, with rigorous journal selection criteria, a comprehensive citation network, and highly standardized bibliographic records (Birkle et al., 2020). Scopus is a broad abstract and citation database that covers a large body of peer-reviewed literature across multiple disciplines (Pasko et al., 2021). The combined use of WoSCC and Scopus helped reduce potential source bias associated with a single database and improved literature coverage and data completeness. All searches were completed on July 3, 2026. The final dataset was obtained through database retrieval, publication type restriction, thematic relevance screening, database merging, and duplicate removal. The detailed literature screening workflow is shown in Figure 1.

**Figure 1:**
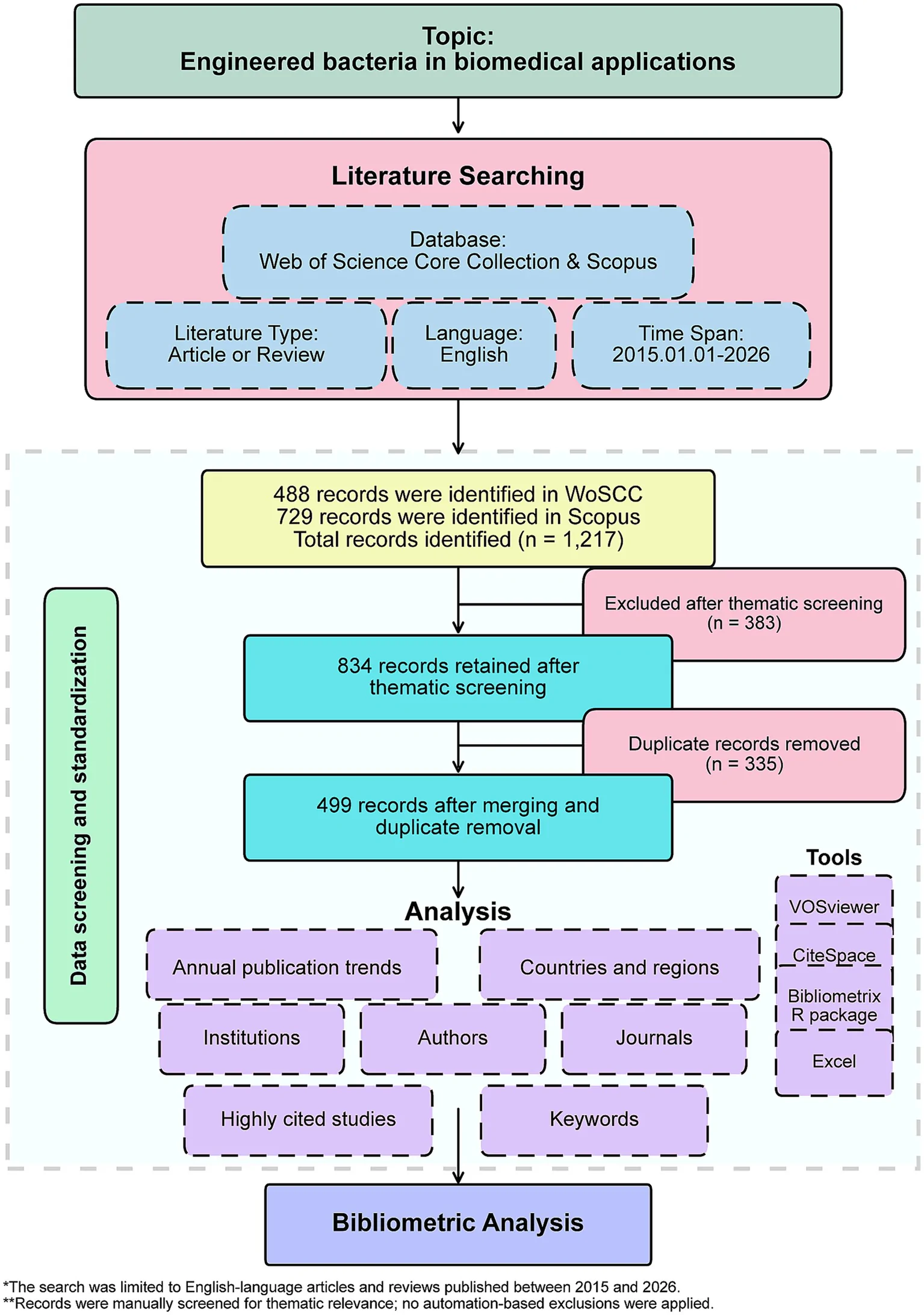
Flow diagram of literature identification, screening, database merging, duplicate removal, and final inclusion.

The search strategy was constructed using three conceptual modules: terms related to engineered bacteria or engineered probiotics, terms related to intestinal diseases, and terms related to therapeutic applications. The first module included terms such as “engineered bacteria,” “genetically engineered bacteria”, “recombinant bacteria”, “engineered probiotics”, “synthetic probiotics”, and “programmable probiotics.” The second module included terms such as “inflammatory bowel disease”, “ulcerative colitis”, “Crohn’s disease”, “colitis”, “intestinal barrier”, “intestinal injury”, “colorectal cancer”, “necrotizing enterocolitis”, “constipation”, and “radiation-induced intestinal damage”. The third module included terms such as “therapy”, “treatment”, “prevention”, “anti-inflammatory”, “immunotherapy”, and “drug delivery”. This mandatory therapeutic-term block was used to improve thematic specificity by limiting retrieval to publications with an explicit therapeutic orientation. However, relevant platform studies that did not use these terms in the indexed fields could have been missed, representing a potential trade-off between retrieval specificity and sensitivity. Searches in WoSCC were conducted using the Topic field, whereas searches in Scopus were conducted using the Title, Abstract, and Keywords fields. Because database syntax and field behavior differ, the database-specific search logic was not fully identical. In WoSCC, an additional NOT block was used to improve specificity by excluding recurrent non-target diseases and applications, whereas an equivalent exclusion block was not applied in Scopus. This discrepancy may have contributed to database-specific retrieval heterogeneity. The complete search strategy is provided in Supplementary Table S1.

English-language articles and reviews published between 2015 and 2026 were included. After database-specific year, language, and document-type restrictions, 488 records were retained from WoSCC and 729 from Scopus. Thematic relevance screening based on titles, abstracts, and keywords resulted in 377 eligible records from WoSCC and 457 from Scopus. These records were subsequently merged, yielding 834 records, of which 335 duplicates were removed. A total of 499 publications were therefore included in the final bibliometric and visualization analyses, comprising 449 original research articles and 50 reviews. Because the search was completed on July 3, 2026, publications from 2026 represented a partial-year dataset.

### 2.2 Data processing and bibliometric analysis

Records from WoSCC were exported in plain text format with “Full Record and Cited References”, whereas records from Scopus were exported in CSV format. The records from both databases were then imported into R and Python environments for data cleaning, field standardization, and database integration. Data preprocessing was performed with reference to the data structure requirements of the bibliometrix package and was further supplemented using customized scripts. For records from WoSCC and Scopus, fields including title, author, publication year, journal, DOI, keywords, and affiliations were first standardized. Duplicate records were primarily identified using DOI information. For records without DOI information or with inconsistent title formats across databases, manual verification was further performed based on standardized titles, publication years, and journal names to reduce duplicate identification bias caused by differences in capitalization, punctuation, abbreviations, and special characters.

The final merged and deduplicated dataset comprised 499 publications. To improve the reliability of author-level analyses, author names were automatically standardized and then manually checked for highly productive authors and names with potential spelling variants, abbreviated forms, or accent-related differences. Keywords, country/region names, and institutional names were merged and standardized when necessary using a thesaurus and manual correction while retaining the original database information. This process was conducted to minimize the influence of synonyms, spelling variants, and database-specific export formats on network analysis results.

Bibliometric analysis and visualization were subsequently performed using R, Python, VOSviewer, and CiteSpace. R and the bibliometrix package were mainly used for descriptive statistical analyses, including annual publication trends and the distribution of countries/regions, institutions, journals, and authors. Python was mainly used for data cleaning, field standardization, and auxiliary figure preparation. VOSviewer was used to construct collaboration networks of countries/regions, institutions, and authors, as well as keyword co-occurrence networks and co-citation networks. CiteSpace was used for co-cited reference clustering, timeline visualization, burst keyword detection, and burst reference analysis. To ensure the clarity and interpretability of network maps, minimum inclusion thresholds were set according to different analytical objectives. For example, countries/regions with at least five publications were included in the country/region collaboration analysis, and keywords with a minimum occurrence frequency of nine were included in the keyword co-occurrence analysis. Unless otherwise specified, the remaining software parameters were set to their default values.

## 3 Results and discussion

### 3.1 Publication trends by year

The annual publication trends of studies on engineered bacteria and engineered probiotics for intestinal disease therapy from 2015 to 2026 are shown in Figure 2. Overall, the number of publications exhibited a clear upward trend. Between 2015 and 2020, the annual output remained relatively stable, with no more than 32 publications per year, suggesting that publication activity in this field remained relatively limited during this period. Since 2021, the number of publications has gradually increased, reaching 40 in 2021 and 42 in 2023, followed by a further rise to 60 in 2024 (Figure 2A). In 2025, the annual output reached 109 publications, the highest value during the study period and 49 more than that in 2024 (Figure 2B), indicating a marked acceleration in publication activity in recent years.

**Figure 2:**
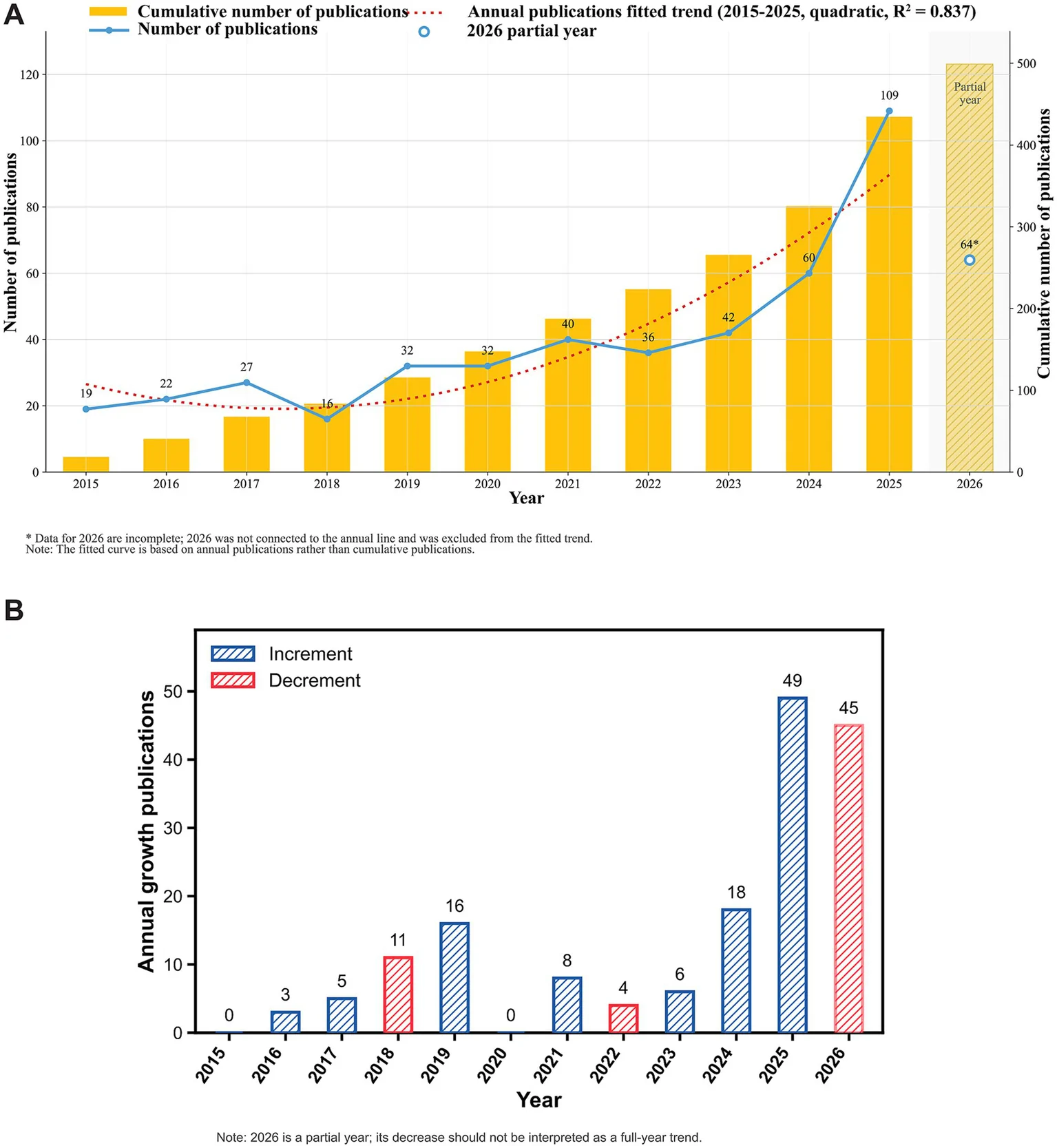
Annual publication trends of engineered bacteria and engineered probiotics for intestinal disease therapy from 2015 to 2026. **(A)** Annual and cumulative publications, with a quadratic fitting curve based on data from 2015 to 2025. Data from 2026 were incomplete and excluded from fitting. **(B)** Year-on-year changes in annual publication output.

A quadratic fitting curve based on annual publication output from 2015 to 2025 showed a good fit (*R*^2^ = 0.837), indicating an accelerating growth trend in publication output on engineered bacteria and engineered probiotics for intestinal disease therapy. The cumulative number of publications also increased continuously over time, reaching 435 by 2025. By the retrieval date, 64 relevant publications had been recorded in 2026. However, because the 2026 dataset was incomplete, this year was not included in the annual trend fitting. Taken together, the annual publication pattern indicates that engineered bacteria and engineered probiotics for intestinal disease therapy have become a rapidly developing research direction, with particularly marked growth during 2024–2025.

Possible factors discussed in the underlying literature that may have contributed to this publication growth include rapid advances in synthetic biology, genetic engineering, and microbiome technologies, which have provided an important technical foundation for bacterial chassis engineering, genetic circuit design, disease microenvironment-responsive systems, and living delivery platforms (Cubillos-Ruiz et al., 2021). In particular, the development of programmable genetic circuits, environment-responsive expression systems, biosafety switches, and engineered probiotic chassis has been associated with increasing research attention to controllable, modular, and precision-oriented living therapeutic platforms (Riglar and Silver, 2018).

Second, the increasing burden of IBD, CRC, and gut dysbiosis-associated diseases has created a growing demand for locally precise, long-lasting, and low-systemic-toxicity therapeutic strategies for intestinal disorders (Zou et al., 2024). Compared with conventional drugs, the underlying literature reports potential advantages of engineered bacteria and engineered probiotics in intestinal colonization, *in situ* production of therapeutic molecules, regulation of microbial homeostasis, and remodeling of the mucosal immune microenvironment, which may have contributed to increasing research attention in intestinal disease therapy (Zhou et al., 2022). Third, increasing integration of engineered bacteria with biomaterials, nanodelivery systems, outer membrane vesicles, and tumor immunotherapy is reflected in a broadening of publication themes from IBD to CRC, intestinal infections, intestinal barrier injury, and other disease contexts (Cheng et al., 2021; Nguyen et al., 2023). With continued research on biosafety control, functional programmability, delivery efficiency, and translational evaluation, this field is likely to remain an active research area.

### 3.2 Collaborative network analysis of engineered bacteria and engineered probiotics for intestinal disease therapy

#### 3.2.1 Collaborative network analysis for countries/regions

Country/region collaboration analysis revealed distinct regional clustering and a multicenter collaboration pattern in research on engineered bacteria and engineered probiotics for intestinal disease therapy. China and the United States were the two leading contributors in this field. China had the highest publication output and occupied a central position in the collaboration network, whereas the United States, despite having fewer publications than China, showed a high total link strength, indicating strong overall international collaboration connectivity (Figure 3A).

**Figure 3:**
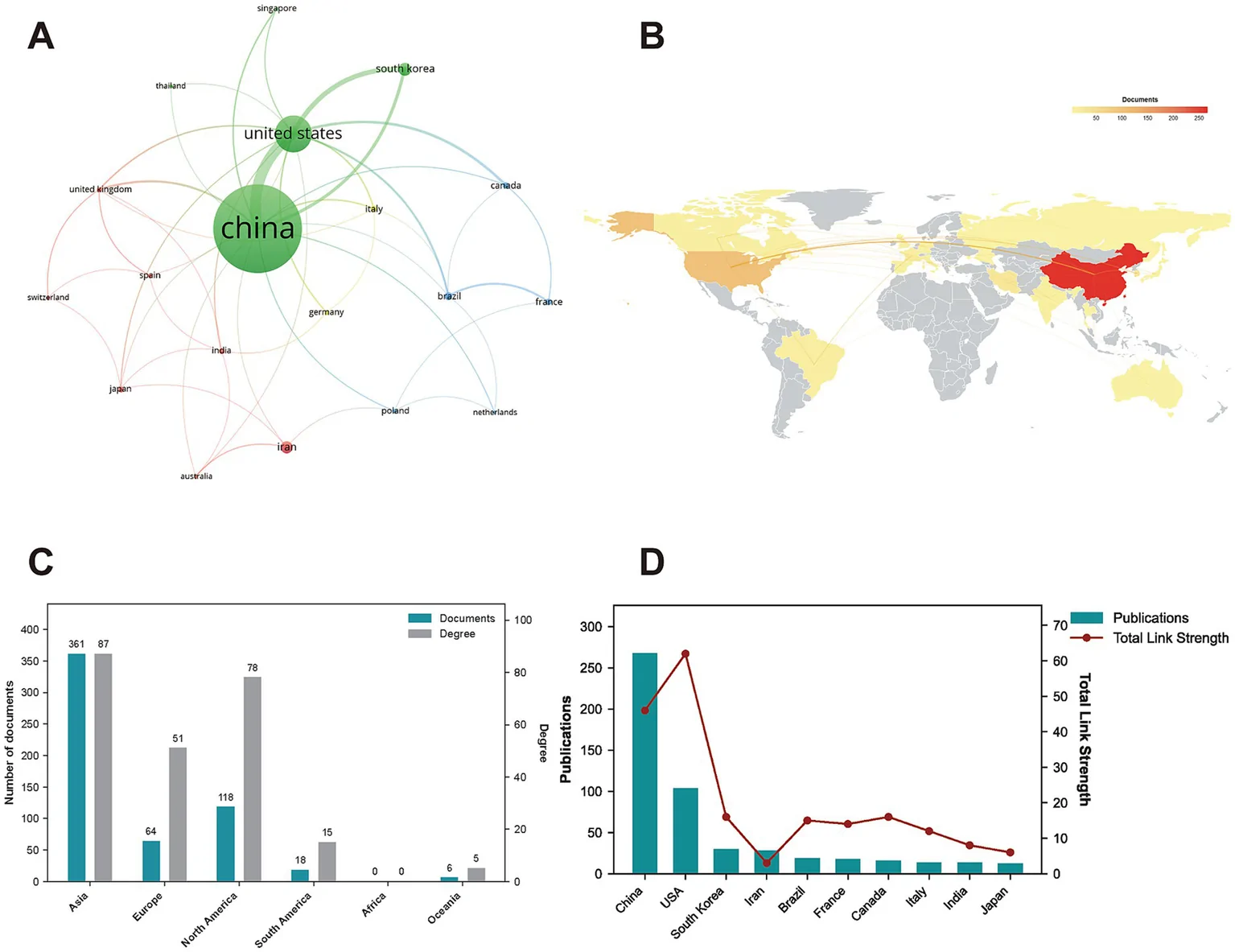
Global distribution and collaboration network of countries/regions in this field. **(A)** Country/region collaboration network; **(B)** global geographic distribution of publications; **(C)** publication output and collaboration connectivity by continent; **(D)** publication output and total link strength of leading countries/regions.

The global distribution map further showed that relevant studies were mainly concentrated in Asia, North America, and Europe, with particularly active contributions from China, the United States, South Korea, Japan, Germany, the United Kingdom, France, India, and Canada (Figure 3B). At the continental level, Asia showed a dominant publication output, contributing 361 publications. Although North America and Europe produced fewer publications than Asia, they exhibited relatively high collaboration connectivity, indicating strong international collaboration capacity and active cross-regional knowledge exchange (Figure 3C). Analysis of publication output and total link strength at the country level further confirmed that China was the most productive country in this field, whereas the United States had the highest total link strength. South Korea, Iran, Brazil, France, Canada, Italy, India, and Japan also represented important contributors to this research area (Figure 3D).

This multiregional participation pattern has important implications for engineered bacterial therapy in intestinal diseases. The composition of the gut microbiota is influenced by multiple factors, including geographic location, dietary habits, lifestyle, and host genetic background. Therefore, contributions from different countries and regions may facilitate the evaluation of engineered bacterial therapeutic strategies across diverse populations and intestinal microbial contexts (Sun et al., 2020). Future studies should further strengthen cross-regional collaboration to systematically assess the safety, colonization capacity, and therapeutic applicability of different bacterial chassis, delivery strategies, and therapeutic payloads across distinct disease spectra, population characteristics, and microbiota backgrounds.

#### 3.2.2 Collaborative network analysis for institutions

A bibliometric analysis of publishing institutions was performed to characterize the institutional collaboration landscape in the field of engineered bacteria and engineered probiotics for intestinal disease therapy. The institutional collaboration network showed several relatively distinct collaborative clusters, with the Chinese Academy of Sciences, Soochow University, Harvard University / Harvard Medical School, Paris-Saclay University, the Jozef Stefan Institute, and the University of Ljubljana serving as major research nodes (Figure 4A). Overall, Chinese institutions occupied prominent positions in the network, indicating their high research output and active institutional participation in this field. Meanwhile, several relatively stable collaborative links were observed among European and North American institutions, reflecting a certain degree of international coordination.

**Figure 4:**
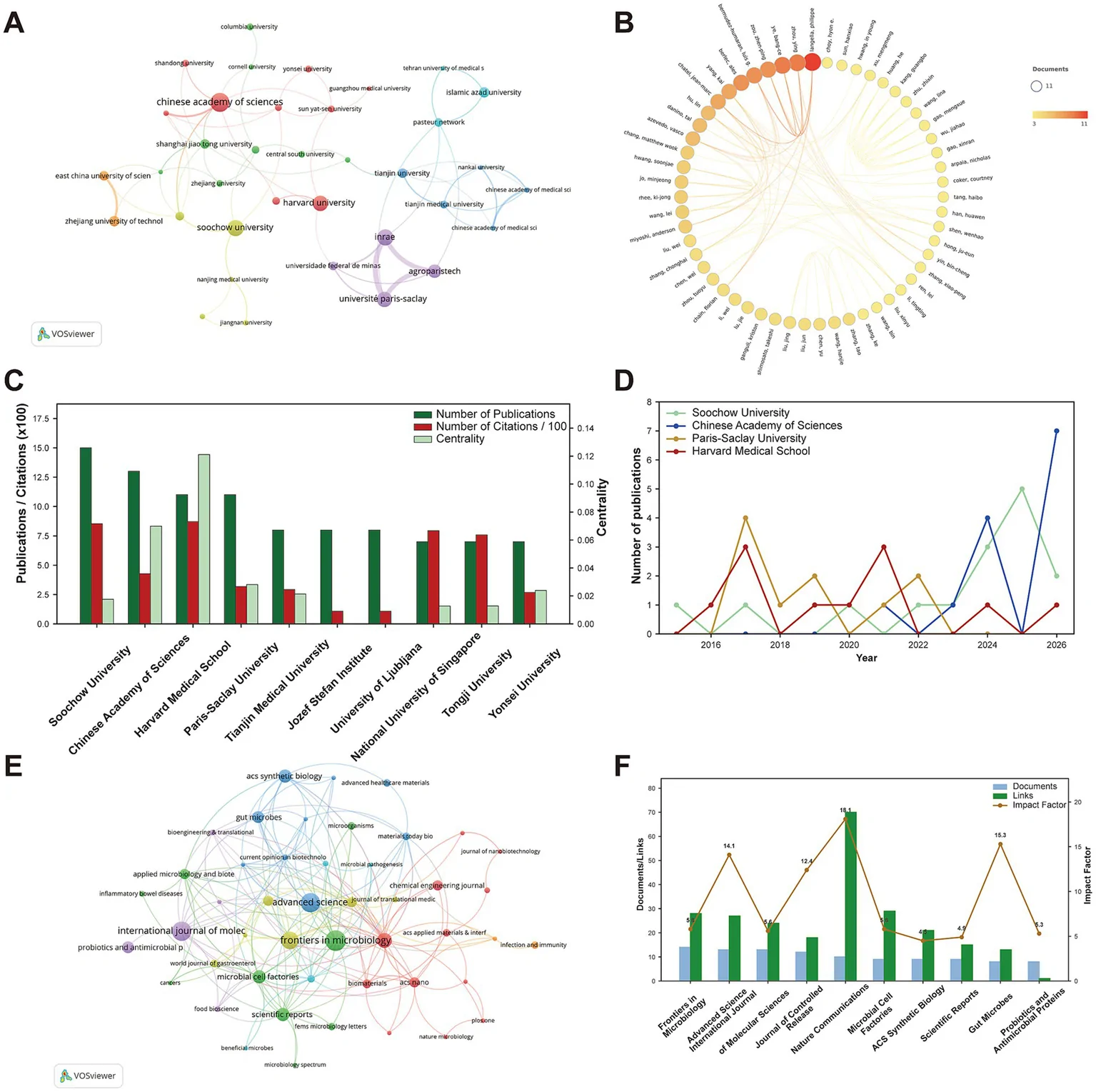
Institutional, author, and journal analyses of engineered bacteria and engineered probiotics for intestinal disease therapy. **(A)** Institutional collaboration network; **(B)** Author collaboration and publication distribution; **(C)** Publication output, citation counts, and centrality of major institutions; **(D)** Annual publication trends of representative institutions; **(E)** Journal co-occurrence network; **(F)** Leading journals ranked by publications, links, and impact factor.

In terms of publication output, citation counts, and centrality metrics, Soochow University, the Chinese Academy of Sciences, Harvard Medical School, Paris-Saclay University, Tianjin Medical University, the Jozef Stefan Institute, the University of Ljubljana, the National University of Singapore, Tongji University, and Yonsei University were identified as important contributing institutions in this field (Figure 4C). Among them, Soochow University and the Chinese Academy of Sciences showed relatively high publication output, suggesting active research productivity. In contrast, institutions such as Harvard Medical School showed stronger citation impact, indicating that some institutions, although not necessarily the most productive, have made highly influential contributions.

The annual publication trajectories of major institutions further revealed differences in their temporal patterns of participation (Figure 4D). Soochow University and the Chinese Academy of Sciences showed a marked increase in publication output in recent years, suggesting the growing involvement of Chinese institutions in research on engineered bacteria and engineered probiotics. Paris-Saclay University and Harvard Medical School, by contrast, showed earlier or more stage-specific contributions, reflecting the important role of European and North American institutions in the early exploration and technological accumulation of this field.

These temporal differences suggest that institutional participation in this field has changed over time rather than being consistently dominated by a fixed set of research centers. Earlier publication activity from European and North American institutions such as Harvard Medical School and Paris-Saclay University may reflect earlier engagement in engineered bacteria, probiotic delivery, and microbial therapeutics, which contributed to the conceptual and technological literature base of the field. In recent years, the rapid increase in publications from Chinese institutions such as Soochow University and the Chinese Academy of Sciences reflects increasing engagement of Chinese research teams in interdisciplinary areas such as synthetic biology, engineered probiotics, biomaterial-assisted delivery, and intestinal disease therapy. Overall, the institutional collaboration landscape shows a temporal shift from relatively dispersed participation toward greater concentration around highly productive institutions with continued multiregional involvement. Given that engineered bacterial therapy for intestinal diseases involves bacterial chassis construction, genetic circuit design, delivery system optimization, disease model validation, and biosafety evaluation, future interdisciplinary collaboration among institutions may support further translational research.

#### 3.2.3 Collaborative network analysis for authors

Author collaboration network analysis further revealed the research team structure and academic collaboration patterns in the field of engineered bacteria and engineered probiotics for intestinal disease therapy. As shown in Supplementary Figure S1, multiple author collaboration clusters were identified. Nodes with different colors represent distinct collaborative groups, node size reflects publication output or collaborative visibility, and links between nodes indicate co-authorship relationships. Overall, the author collaboration network exhibited a clear team-based structure, whereas connections among different clusters were relatively limited. This pattern suggests that the field is currently characterized by the parallel development of multiple research teams rather than a highly integrated global author collaboration network.

In particular, the red cluster centered on Langella, Philippe was relatively concentrated and showed close collaboration with authors such as Chatel, Jean-Marc and Azevedo, Vasco. The purple cluster represented by Zhou, Ying, Zou, Zhen-ping, and Ye, Bang-ce also formed a relatively stable collaborative team. In addition, authors such as Berlec, Ales, Danino, Tal, Yang, Kai, and Hwang, Soonjae showed notable visibility in the network, representing different research directions or regional collaborative groups.

Figure 4B further illustrates the publication contributions and collaborative links of major authors. Consistent with the network structure shown in Supplementary Figure S1, highly productive authors were mainly distributed across several core collaborative groups, whereas direct collaboration among different groups remained insufficient. These findings indicate that, although engineered bacteria and engineered probiotics for intestinal disease therapy have attracted participation from multiple research teams, the author-level collaboration network is still in a developing stage. Overall, the collaboration pattern in this field can be summarized as parallel development among multiple teams, strong intra-team collaboration, and relatively limited inter-team collaboration.

This collaboration pattern may be related to the highly interdisciplinary nature of this research field. Engineered bacterial therapy for intestinal diseases typically involves multiple technical components, including microbial chassis construction, genetic circuit design, therapeutic molecule expression, delivery system development, animal model validation, and biosafety evaluation. Different research teams often focus on specific modules according to their technical strengths, which may lead to the formation of relatively independent and specialized collaborative groups. It should also be noted that author collaboration network analysis has inherent limitations related to author name disambiguation. Although highly frequent author names and potential spelling variants were manually checked in this study, differences in name abbreviations, accent marks, spelling formats, and homonymous authors across databases may still lead to underestimation of publication output or collaborative relationships for certain authors. In the future, integrating ORCID information, institutional affiliations, and more refined author disambiguation algorithms may further improve the accuracy of author collaboration network analysis.

### 3.3 Journal publication analysis and dual-map overlay

The journal collaboration and thematic distribution network showed that studies on engineered bacteria and engineered probiotics for intestinal disease therapy were mainly published in journals related to microbiology, synthetic biology, biomaterials, drug delivery, and gut microbial ecology (Figure 4E). Frontiers in Microbiology, Advanced Science, International Journal of Molecular Sciences, Journal of Controlled Release, Nature Communications, Microbial Cell Factories, ACS Synthetic Biology, Scientific Reports, Gut Microbes, and Probiotics and Antimicrobial Proteins were the major publication sources in this field (Figure 4F). These journals cover multiple disciplines, including basic microbiology, engineering biology, drug delivery, biomaterials, gut microbiota research, and translational medicine, indicating a clear interdisciplinary publication pattern in this research area.

The distribution of source journals further reflects the disciplinary structure of this field. The presence of journals such as Frontiers in Microbiology, Microbial Cell Factories, and ACS Synthetic Biology indicates that microbial engineering, chassis modification, and synthetic biology are prominent components of the publication landscape. The high visibility of journals such as Journal of Controlled Release and Advanced Science suggests increasing publication activity related to drug delivery, biomaterials, and living therapeutic platform construction. Meanwhile, the involvement of Gut Microbes and Probiotics and Antimicrobial Proteins highlights the close relationship between this field and gut microbial ecology, probiotic intervention, and host–microbe interactions. Overall, journal source analysis indicates a shift in publication themes from traditional probiotics and gut microbiota research toward an interdisciplinary area integrating synthetic biology, material-assisted delivery, immune modulation, and translational medicine.

To further reveal the disciplinary distribution and knowledge flow of this field, a dual-map overlay of journals was generated using CiteSpace. In Figure 5, the left side represents the disciplinary domains of citing journals, whereas the right side represents the disciplinary domains of cited journals. The curves indicate citation pathways, and thicker curves represent stronger knowledge linkages between corresponding disciplines. Overall, the citing journals were mainly distributed in the domains of molecular/biology/immunology, medicine/medical/clinical, ecology/earth/marine, and physics/materials/chemistry, suggesting that studies in this field involve microbiology, immunology, clinical medicine, materials science, and drug delivery. The cited journals were mainly distributed in molecular/biology/genetics, health/nursing/medicine, chemistry/materials/physics, and environmental/toxicology/nutrition, indicating that the knowledge base of this field primarily derives from molecular biology, genetics, medicine, materials chemistry, and nutrition- or environment-related microbiology.

**Figure 5:**
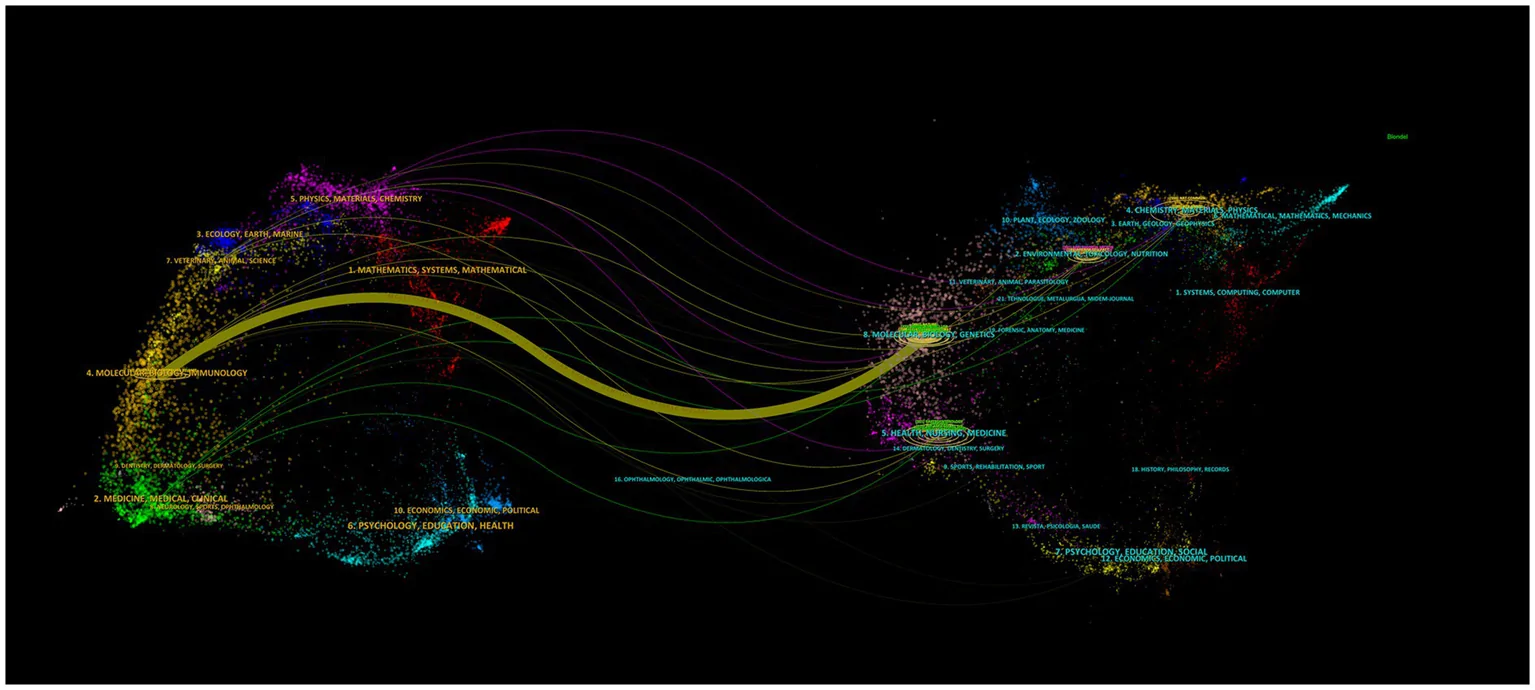
Dual-map overlay of journals in engineered bacteria and engineered probiotics for intestinal disease therapy. The left and right panels represent the disciplinary distributions of citing and cited journals, respectively, while colored curves indicate major citation pathways between disciplines.

In terms of citation pathways, the most prominent trajectory extended from the molecular/biology/immunology domain on the citing side to the molecular/biology/genetics domain on the cited side. This indicates that current research strongly relies on foundational knowledge from molecular biology, microbial genetics, immune regulation, and synthetic biology. In addition, citation pathways from medicine/medical/clinical to health/nursing/medicine and molecular/biology/genetics were also evident, suggesting that this field is not only focused on mechanistic research but is also increasingly connected with intestinal disease therapy, translational research, and disease model studies. The presence of materials- and chemistry-related pathways further indicates that biomaterials, nanoscale delivery systems, and engineered carriers provide important technical support for engineered bacterial therapeutic strategies.

Taken together, the journal source distribution and dual-map overlay analysis indicate that engineered bacteria and engineered probiotics for intestinal disease therapy constitute a multidisciplinary research field. Its knowledge structure is supported by literature from microbiology, immunology, molecular genetics, clinical medicine, materials science, and drug delivery. These findings further indicate a shift in publication themes from traditional probiotics and gut microbiota research toward interdisciplinary topics involving synthetic biology, living therapeutics, material-assisted delivery, and clinical translation.

### 3.4 Keyword analysis and clustering of engineered bacteria and engineered probiotics for intestinal disease therapy

Keyword co-occurrence analysis is an effective approach for identifying core research hotspots and tracking changes in research attention. As shown in Figure 6A, the keyword co-occurrence network formed several high-frequency nodes centered on inflammatory bowel disease probiotics gut microbiota engineered probiotics engineered bacteria synthetic biology colitis and intestinal barrier. Among these inflammatory bowel disease probiotics and gut microbiota were located at the center of the network with large node sizes and strong connections to other keywords indicating that they represent highly connected core themes in the bibliometric network. Previous studies have shown that the pathogenesis and progression of IBD are closely associated with gut microbial dysbiosis epithelial barrier dysfunction and disruption of mucosal immune homeostasis while probiotic and microbiota-modulating strategies are considered important approaches for improving the intestinal inflammatory microenvironment (Vindigni et al., 2016; Shan et al., 2021). Meanwhile keywords such as engineered probiotics engineered bacteria genetic engineering and synthetic biology showed strong associations with these core thematic nodes indicating increasing research attention to synthetic biology-driven engineered bacterial therapeutics. The underlying literature reports that synthetic biology and genetic circuit design can enable engineered bacteria to sense intestinal inflammation oxidative stress hypoxia or specific metabolic signals and to express or release therapeutic molecules locally at diseased sites (Huang et al., 2022)

**Figure 6:**
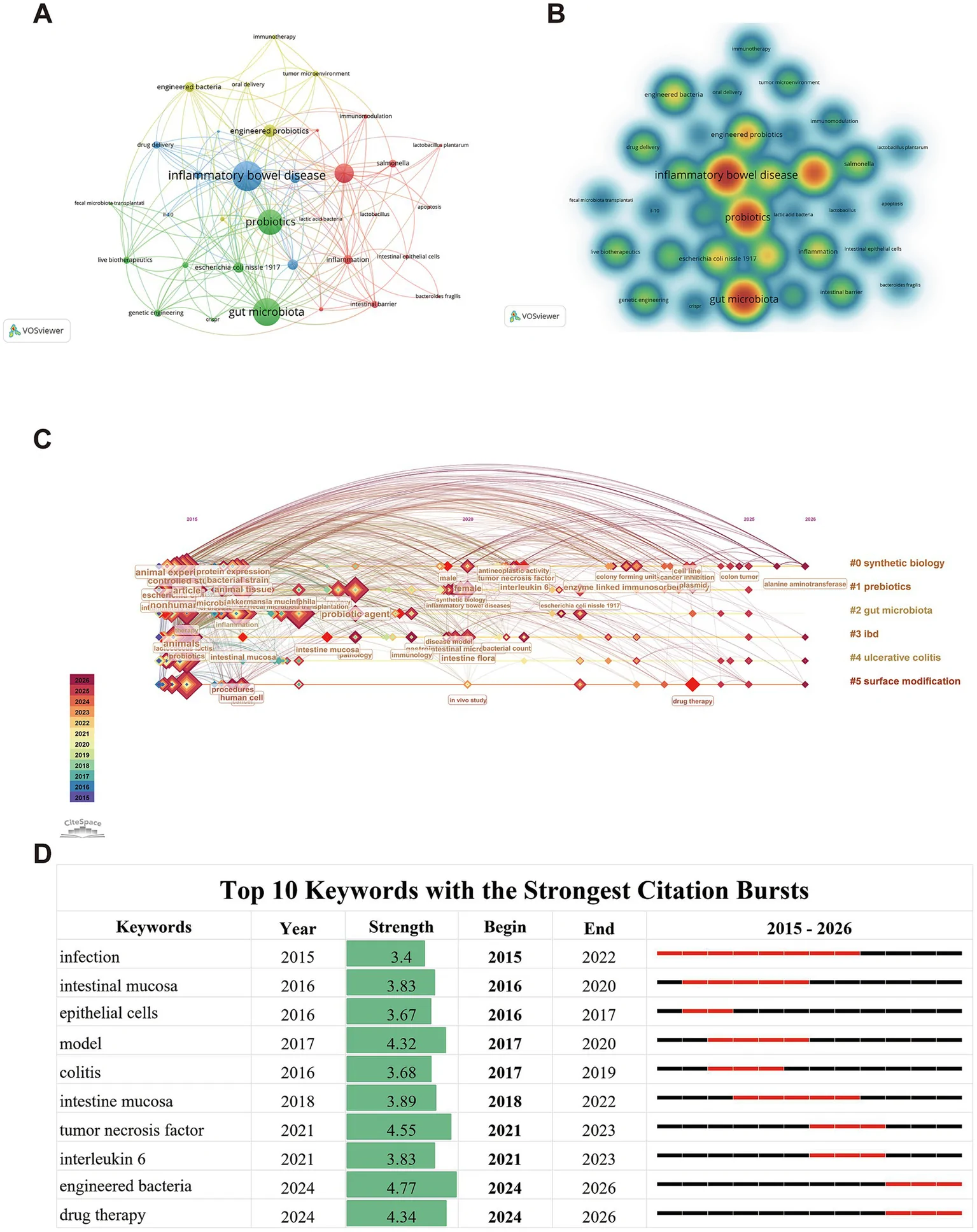
Keyword analysis of engineered bacteria and engineered probiotics for intestinal disease therapy. **(A)** Keyword co-occurrence network; **(B)** Keyword density visualization; **(C)** Timeline visualization of major keyword clusters; **(D)** Top 10 keywords with the strongest citation bursts.

The keyword density map further supported these findings (Figure 6B). High-density regions were mainly concentrated around inflammatory bowel disease, probiotics, gut microbiota, and engineered probiotics, indicating that these topics represent highly active and accumulated research hotspots. In addition to IBD-related research, keywords such as colorectal cancer, tumor microenvironment, immunotherapy, and drug delivery also appeared in the network, indicating that publication themes extend beyond intestinal inflammation to include CRC therapy, tumor microenvironment research, and drug delivery. In the underlying literature, engineered bacteria have been reported to exploit hypoxic, necrotic, and immunosuppressive features of solid tumors for selective colonization and to enhance antitumor immune responses by releasing immunomodulatory factors, nanobodies, or tumor antigens (Nguyen et al., 2023). In parallel, material encapsulation, outer membrane vesicles, and local delivery platforms have been investigated to improve bacterial stability, delivery efficiency, and biosafety (Sang et al., 2025).

CiteSpace timeline analysis showed that the major keyword clusters in this field included synthetic biology, prebiotics, gut microbiota, IBD, ulcerative colitis, and surface modification (Figure 6C). From a temporal perspective, earlier publications were more frequently associated with topics related to basic disease models and intestinal mucosal injury, such as infection, intestinal mucosa, epithelial cells, model, and colitis. Subsequently, research attention became more concentrated on microbiota regulation and IBD-related topics, including gut microbiota, IBD, and ulcerative colitis. In recent years, keywords such as synthetic biology, engineered bacteria, and surface modification have become increasingly active, suggesting increasing research attention to bacterial chassis design, surface modification, synthetic biology-based regulation, and living delivery systems.

Burst keyword analysis further revealed time-dependent changes in research attention (Figure 6D). During 2015–2022, infection and intestinal mucosa showed sustained bursts, indicating continued attention to infection-associated intestinal injury and mucosal barrier-related topics. From 2017 to 2020, burst keywords such as model and colitis appeared, indicating the prominence of experimental models and colitis-related research during this period. During 2021–2023, tumor necrosis factor and interleukin 6 emerged as burst keywords, reflecting increasing attention to inflammatory cytokines and immunoinflammatory mechanisms. Notably, engineered bacteria and drug therapy showed sustained bursts during 2024–2026, with engineered bacteria exhibiting the strongest burst intensity, indicating increasing publication attention to engineered bacterial therapeutics in the recent literature.

Overall, keyword analysis indicates a temporal shift in research attention from intestinal infection and mucosal injury toward IBD, gut microbiota regulation, and inflammatory cytokine-related topics, with more recent emphasis on synthetic biology, engineered bacterial design, and drug delivery-based therapeutic platforms. Current research hotspots include precise local intestinal delivery, inflammatory microenvironment responsiveness, immune modulation, and tumor microenvironment-related applications. These bibliometric patterns suggest increasing attention to programmable, controllable, and multifunctional living therapeutic systems and to engineering optimization focused on precision, safety, and controllability.

### 3.5 Co-cited reference analysis and temporal shifts in research frontiers

Co-cited reference analysis can reveal the knowledge base and key intellectual trajectories of engineered bacteria and engineered probiotics for intestinal disease therapy. As shown in Figure 7, the co-citation network formed several relatively distinct clusters, suggesting that the literature base of this research area is associated with engineered probiotics, synthetic biology, intestinal inflammation therapy, and bacteria-mediated cancer therapy. Figure 7A shows that several classic studies occupy central positions in the network, including early work on engineered *Lactococcus lactis* for the delivery of anti-inflammatory factors (Steidler et al., 2000), as well as more recent studies on engineered *Escherichia coli* Nissle 1917, programmable bacterial therapeutics, and bacteria-mediated antitumor immunity. Together, these highly co-cited studies form an important knowledge base associated with the increasing prominence of programmable living therapeutic strategies (Riglar and Silver, 2018).

**Figure 7:**
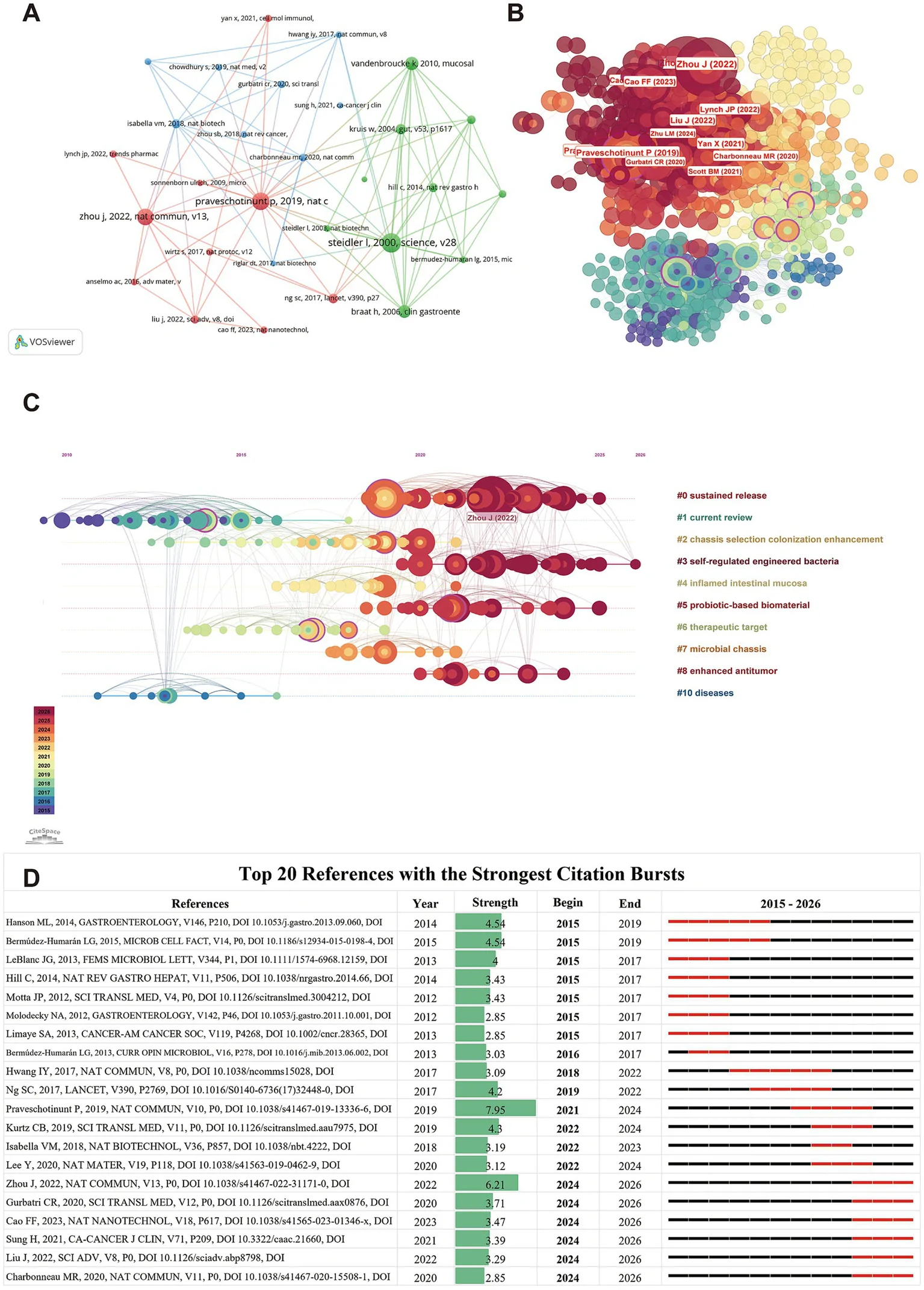
Co-cited reference analysis of engineered bacteria and engineered probiotics for intestinal disease therapy: **(A)** Co-citation network of references; **(B)** Co-citation clustering map; **(C)** Timeline visualization of major co-citation clusters; **(D)** Top 20 references with the strongest citation bursts.

The clustering structure of co-cited references further revealed clear thematic differentiation within the field (Figure 7B). Earlier clusters were mainly associated with probiotics, intestinal mucosal immunity, inflammatory bowel disease, and intestinal barrier repair. In contrast, more recent high-density regions were more closely related to engineered bacterial chassis, synthetic biology-based regulation, drug delivery, the tumor microenvironment, and immunotherapy. These findings indicate that the thematic emphasis of the literature extends beyond conventional probiotic anti-inflammatory research and increasingly includes genetic circuit design, disease microenvironment responsiveness, living delivery, and immune modulation.

The timeline visualization further illustrated temporal shifts in thematic emphasis within the knowledge structure of this field (Figure 7C). The major clusters included sustained release, chassis selection and colonization enhancement, self-regulated engineered bacteria, inflamed intestinal mucosa, probiotic-based biomaterial, microbial chassis, and enhanced antitumor. Earlier publications were more frequently associated with intestinal inflammation, mucosal barrier function, and probiotic therapy. With advances reported in synthetic biology and microbial engineering, research attention increasingly included microbial chassis selection, enhanced intestinal colonization, self-regulated engineered bacteria, and probiotic-based biomaterials (Din et al., 2016). In recent years, clusters such as sustained release, probiotic-based biomaterial, and enhanced antitumor have remained active, suggesting continued or increasing research attention to sustained delivery systems, biomaterial-assisted engineered bacterial therapy, and engineered bacteria-mediated antitumor therapy (Sang et al., 2025).

From a temporal perspective, three broad thematic emphases can be observed, although these should not be interpreted as strictly separated or linear developmental stages. Earlier literature emphasized delivery of anti-inflammatory factors by engineered probiotics and intestinal mucosal inflammation. Subsequently, synthetic biology concepts became more prominent, and publication themes increasingly included sensing of disease-associated signals, regulated expression, and controllable release. More recent literature has placed greater emphasis on delivery efficiency, biosafety, disease adaptability, biomaterial encapsulation, outer membrane vesicles, sustained-release systems, tumor-targeting engineered bacteria, and immunotherapeutic payloads. This temporal pattern suggests a shift in research attention toward more modular and translationally oriented platform design rather than a definitive linear evolution of the field.

Burst reference analysis further identified representative key studies across different time periods (Figure 7D). Early burst references were mainly associated with the definition of probiotics, engineered probiotic delivery of anti-inflammatory factors, and regulation of intestinal inflammation, indicating that the early knowledge base of this field was largely built upon probiotic therapy and mucosal immune modulation. After 2019, burst references became increasingly concentrated on engineered *E. coli* Nissle 1917, synthetic biology-driven living therapeutic platforms, and bacteria-mediated cancer immunotherapy. Some of these references continued to show citation bursts until 2026, indicating their sustained influence on current research development.

Notably, the co-citation clusters and burst references together suggest that recent research attention increasingly emphasizes precision, stability, and safety in addition to therapeutic feasibility. For example, clusters related to microbial chassis selection and colonization enhancement reflect attention to stable function in the complex intestinal environment, whereas self-regulated engineered bacteria and disease microenvironment-responsive systems reflect interest in the spatiotemporal control of therapeutic molecule release. Probiotic-based biomaterials and sustained-release systems are associated with research on gastrointestinal delivery, local retention, and therapeutic durability. In addition, the activity of the enhanced antitumor cluster indicates increasing research attention to CRC and tumor immune regulation. These patterns support continued integration of chassis engineering, delivery materials, genetic circuits, biosafety switches, and disease model validation in future research.

Overall, co-cited reference and burst reference analyses indicate increasing prominence of research themes involving engineered bacterial chassis design, programmable genetic circuits, sustained local intestinal delivery, and disease microenvironment responsiveness relative to earlier emphasis on conventional probiotic therapy. This bibliometric pattern reflects changes in thematic attention and should not be interpreted as a strictly linear technological progression or as direct evidence of therapeutic superiority.

### 3.6 Conceptual synthesis of bibliometrically identified therapeutic themes

Based on the results of keyword co-occurrence, burst keyword, and co-cited reference analyses, we constructed a conceptual synthesis of major therapeutic themes reported in the underlying literature (Figure 8). The framework organizes bibliometrically identified topics into recurrent research themes, including disease microenvironment responsiveness, delivery of anti-inflammatory factors or therapeutic molecules, intestinal epithelial barrier repair, gut microbiota remodeling, local drug delivery, and modulation of the tumor immune microenvironment. It is intended to summarize thematic relationships and literature-reported therapeutic strategies rather than to represent a mechanistic model directly established or experimentally validated by the present bibliometric analysis.

**Figure 8:**
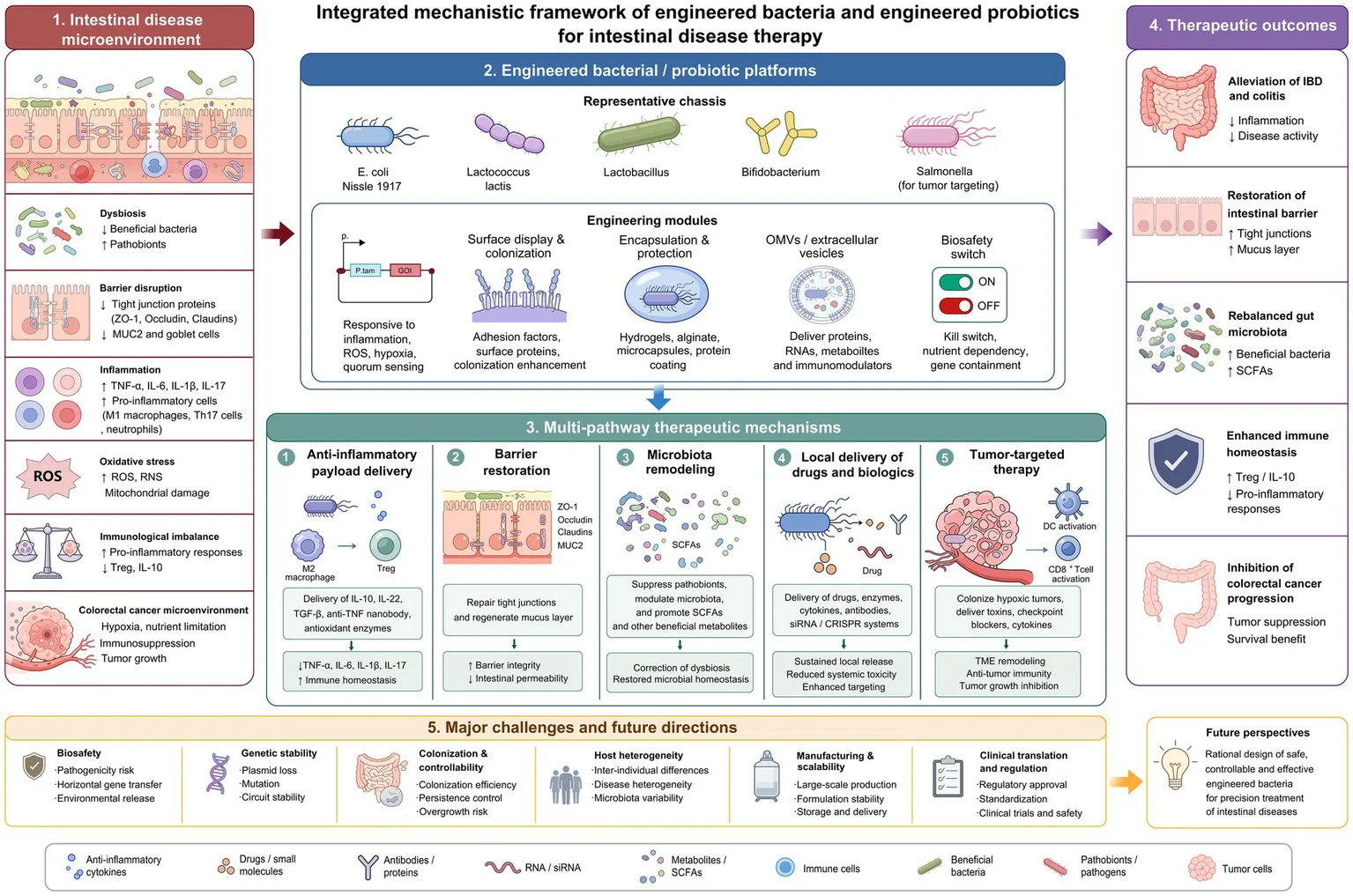
Conceptual synthesis of bibliometrically identified therapeutic themes in engineered bacteria and engineered probiotics for intestinal disease therapy. The framework organizes major themes identified through bibliometric analyses together with representative therapeutic strategies reported in the underlying literature, including disease-related intestinal microenvironmental alterations, engineered bacterial/probiotic platforms, major engineering modules, reported therapeutic strategies and biological processes, application-related outcomes, and key challenges for future translation. It should not be interpreted as a mechanistic model directly established by the present bibliometric study.

Within the underlying literature, disease microenvironment responsiveness represents a recurrent therapeutic design theme. Intestinal diseases are commonly accompanied by elevated inflammatory cytokines, increased oxidative stress, mucosal barrier disruption, microbial dysbiosis, and alterations in the local metabolic environment. In tumor-associated intestinal diseases such as CRC, the pathological microenvironment is also characterized by hypoxia, necrosis, and immunosuppression. These pathological signals provide a basis for the targeted design and selective functional activation of engineered bacteria. Through synthetic biology and genetic circuit design, engineered bacteria can be endowed with the ability to sense inflammation, reactive oxygen species (ROS), hypoxia, quorum-sensing molecules, or specific metabolites, and to selectively initiate therapeutic molecule expression or release at diseased sites, thereby enabling disease microenvironment-responsive therapy (Weibel et al., 2024; Yuan et al., 2025; Niu et al., 2026).

A second recurrent theme in the underlying literature is the local delivery of anti-inflammatory factors and therapeutic proteins for IBD and colitis. Engineered chassis such as *Lactococcus lactis*, *Escherichia coli* Nissle 1917, and Lactobacillus can be designed to locally express or secrete IL-10 (Steidler et al., 2000), IL-22 (Lin et al., 2017), anti-TNF nanobodies (Vandenbroucke et al., 2010), antioxidative enzymes (Zhou et al., 2022), cytokines, or other immunomodulatory molecules (Namai et al., 2020). Reported studies suggest that this strategy can confine therapeutic molecules to the intestinal site, thereby increasing local effective concentrations while reducing systemic exposure and potential adverse effects. In the cited literature, such platforms have been reported to modulate proinflammatory mediators and immune-cell responses, with associated improvements in intestinal inflammatory status. More recently, an engineered *Escherichia coli* Nissle 1917 strain secreting an anti-TNF-α nanobody was shown to achieve localized TNF-α neutralization and alleviate DSS-induced colitis, further supporting the use of engineered probiotic chassis for local delivery of biologically active therapeutic proteins (Zhu Q. et al., 2026).

A third recurring theme in the underlying literature is intestinal epithelial barrier repair. Intestinal diseases are frequently associated with reduced tight junction proteins, disruption of the mucus layer, impaired goblet cell function, and increased intestinal permeability. Reported engineered bacterial strategies aim to promote the restoration of barrier-associated molecules, such as ZO-1, occludin, claudins, and MUC2, by delivering pro-repair molecules, anti-inflammatory factors, metabolic modulators, or matrix-like structures. These reported effects may improve mucosal integrity and reduce the translocation of pathogen-associated molecules and inflammatory mediators across the epithelial barrier (Teng et al., 2022). The sustained appearance of keywords such as intestinal barrier, intestinal mucosa, and epithelial cells is consistent with continued research attention to barrier-related strategies.

Gut microbiota remodeling and metabolic regulation represent another recurrent theme in the underlying literature. Engineered probiotics have been reported both as delivery vehicles for therapeutic molecules and as modulators of the intestinal ecosystem. Reported approaches include competitive colonization, enhanced mucosal adhesion, inhibition of pathogen colonization, regulation of microbial community composition, and modulation of microbiota-derived metabolites, including short-chain fatty acids, bile acids, and tryptophan metabolites (Bai and Mansell, 2020; Drolia et al., 2020; Kim et al., 2021; Gong et al., 2023; Guo et al., 2024). The appearance of keywords such as gut microbiota, probiotics, prebiotics, and SCFAs in the keyword network indicates that gut microbial regulation remains a prominent research theme. In the underlying literature, controllable genetic circuits have also been investigated as a means of improving functional stability and disease adaptability.

The underlying literature also describes engineered bacteria as local intestinal delivery platforms for drugs and biomacromolecules. Reported platform characteristics include living proliferation, local colonization, *in situ* expression, and sustained release. These properties have been explored for the delivery of small-molecule drugs, antibody fragments, enzymes, cytokines, nucleic acids, or gene therapy-related components (Praveschotinunt et al., 2019; Chua et al., 2023; Sahoo et al., 2025). In recent years, biomaterials, microcapsules, hydrogels, nanoscale delivery systems, and outer membrane vesicles have been introduced into engineered bacterial therapeutic systems to improve gastrointestinal stability, intestinal retention, therapeutic payload release efficiency, and biosafety (Cook et al., 2012; Sang et al., 2025). This is consistent with co-citation clusters such as sustained release, probiotic-based biomaterial, and surface modification, indicating that delivery system optimization has become an important research theme. Recent single-cell encapsulation of *Agathobacter rectalis* using an *Aloe vera*-based coating further demonstrated that material-assisted microbial delivery can enhance gastric survival, intestinal colonization, and therapeutic efficacy in experimental colitis (Li et al., 2026).

Finally, in CRC-related literature, engineered bacteria have been investigated for their ability to exploit the hypoxic, necrotic, and immunosuppressive characteristics of solid tumors to achieve selective enrichment. Through controllable lysis systems or genetic circuits, they can locally release immunomodulatory molecules, nanobodies, tumor antigens, or other therapeutic payloads within tumors (Chowdhury et al., 2019; Gurbatri et al., 2020). Reported strategies include direct inhibition of tumor cell growth, activation of antigen presentation, enhancement of CD8^+^ T cell-mediated antitumor immune responses, and remodeling of the immunosuppressive tumor microenvironment. The appearance of topics such as colorectal cancer, tumor microenvironment, immunotherapy, and enhanced antitumor in the keyword and co-citation analyses further indicates increasing research attention to tumor immunotherapy in addition to intestinal inflammatory diseases.

Overall, Figure 8 should be viewed as a conceptual synthesis of literature-reported therapeutic strategies associated with bibliometrically identified themes, rather than as evidence that these mechanisms were directly established by the present analysis. The underlying literature frequently describes programmability, sustained local activity, and multifunctional integration as potential advantages of engineered bacterial therapeutics, while the prominence of related keywords and co-citation clusters indicates increasing research attention to these features. Several key challenges also remain prominent in the literature, including stable colonization in the complex intestinal environment, spatiotemporal controllability of therapeutic functions, genetic stability, risks of horizontal gene transfer, immune safety, interindividual microbiota variability, and unclear clinical regulatory pathways. Future research may therefore continue to integrate chassis optimization, genetic circuit design, material-based delivery systems, biosafety switches, and standardized disease model validation.

Despite substantial progress in preclinical studies, the clinical translation of engineered bacterial therapeutics remains relatively limited. More recently, the broader live biotherapeutic product (LBP) field has progressed into Phase 2 evaluation; for example, MH002, a six-strain wild-type commensal consortium, is being evaluated in a randomized Phase 2 trial in patients with mild-to-moderate ulcerative colitis (ClinicalTrials.gov, 2026; NCT07296315). Notably, such wild-type consortia are not genetically engineered and therefore do not fully address the additional regulatory burden associated with engineered strains. For LBPs that fall within its scope, the U.S. Food and Drug Administration has issued specific guidance regarding chemistry, manufacturing, and control information for early clinical trials (U.S. Food and Drug Administration, 2016). In parallel, biosafety assessment of organisms containing recombinant or synthetic nucleic acid molecules can be informed by risk-group and containment frameworks such as the NIH Guidelines, which consider the risk group of the parental agent together with transgene function, pathogenicity, transmissibility, environmental persistence, and potential changes in phenotype when determining appropriate containment (National Institutes of Health, 2024). For engineered bacterial therapeutics, additional concerns include genetic stability, horizontal gene transfer, antimicrobial resistance markers, bacterial shedding, environmental persistence, and unintended host–microbiota interactions. Accordingly, robust biocontainment strategies, standardized manufacturing, and long-term safety evaluation will be critical for advancing engineered bacterial therapeutics toward clinical application.

Several limitations of this study should be acknowledged. First, although the combined use of WoSCC and Scopus improved literature coverage and reduced the source bias associated with a single database, relevant publications indexed exclusively in other biomedical databases, such as PubMed/MEDLINE and Embase, may not have been captured. Moreover, differences in database coverage, indexing policies, and metadata structures may influence bibliometric results. Future studies integrating additional databases may therefore provide further validation and complementary coverage of the present findings. Second, the mandatory therapeutic-term block may have reduced retrieval sensitivity by missing relevant platform studies without explicit therapeutic terms in the indexed fields. In addition, the broad WoSCC NOT block could have excluded otherwise relevant records when exclusion terms appeared incidentally, while the absence of an equivalent exclusion block in Scopus may have introduced database-specific retrieval heterogeneity.

## 4 Conclusion

Based on the Web of Science Core Collection and Scopus databases, this study conducted a bibliometric and visualization analysis of 499 publications on engineered bacteria and engineered probiotics for intestinal disease therapy published between 2015 and 2026. The findings characterized the research landscape, knowledge base, hotspot topics, and temporal changes in thematic emphasis in this field. Annual publication output increased rapidly overall, particularly in the past 2 years, indicating growing research activity in this interdisciplinary area. China and the United States were the leading contributors, while Asia, North America, and Europe constituted the major research regions. Analyses of journals, disciplines, and collaboration networks further showed that the publication landscape integrates microbiology, synthetic biology, drug delivery, biomaterials, gut microbial ecology, and tumor immunology.

Keyword and co-cited reference analyses indicated a shift in research attention from conventional probiotics gut microbiota inflammatory bowel disease and intestinal mucosal barrier research toward engineered bacterial chassis design synthetic biology-based regulation disease microenvironment responsiveness sustained delivery systems and tumor immunotherapy. Rather than demonstrating therapeutic superiority or a definitive linear transition these patterns show that programmable living therapeutic strategies are receiving increasing research attention. Environmental sensing local release sustained delivery and multifunctional design features are capabilities reported in the underlying literature and represented within the thematic structure identified by the present bibliometric analysis.

Overall, the bibliometric evidence maps an expanding and increasingly interdisciplinary research landscape for engineered bacteria and engineered probiotics in intestinal diseases, including inflammatory bowel disease, colorectal cancer, intestinal infections, and intestinal barrier injury. Future research attention is likely to remain focused on bacterial chassis optimization, genetic circuit design, biosafety control, delivery system integration, long-term colonization stability, and translational evaluation. These findings should be interpreted as trends in research activity, thematic emphasis, and capabilities reported in the underlying literature rather than as direct evidence of therapeutic efficacy. This study provides a quantitative reference for understanding the knowledge structure and emerging research directions of the field.

## Data Availability

The data supporting the findings of this study are derived from publicly available records in Web of Science Core Collection and Scopus. The complete search strategy is provided in Supplementary Table S1.
