# Supplementary material for: Engineered bacteria and engineered probiotics for intestinal disease therapy: a bibliometric and knowledge mapping analysis
Source: Front Microbiol. 2026 Sep 24;17:1970221. doi: 10.3389/fmicb.2026.1970221 (PMC13646158; doi:10.3389/fmicb.2026.1970221)
Supplement: Supplementary file 1 [file Supplementary_file_1.DOCX]

Supplementary Material

Engineered bacteria and engineered probiotics for intestinal disease therapy: a bibliometric and knowledge mapping analysis

**Contents:**

**Table S1.** Search strategies used in the Web of Science Core Collection and Scopus databases

**Figure S1.** Author collaboration network

**Table S1.** Search strategies used in the Web of Science Core Collection and Scopus databases

| **Database** | **Search field** | **Complete search strategy** | **Limits** | **Records after database-specific restrictions** |
| --- | --- | --- | --- | --- |
| **Web of Science Core Collection (WoSCC)** | Topic (TS) | TS=(("engineered bacteri*" OR "genetically engineered bacteri*" OR "genetically modified bacteri*" OR "recombinant bacteri*" OR "engineered probiotic*" OR "genetically engineered probiotic*" OR "genetically modified probiotic*" OR "recombinant probiotic*" OR "synthetic probiotic*" OR "programmable probiotic*" OR "designer probiotic*" OR "smart probiotic*" OR "engineered commensal*" OR (("Escherichia coli Nissle" OR "E. coli Nissle" OR EcN OR "Lactococcus lactis" OR Lactobacillus OR Bifidobacterium OR Salmonella OR Bacteroides) NEAR/5 (engineer* OR recombinant OR modified OR synthetic OR programmable OR express* OR secret*))) AND ("inflammatory bowel disease" OR IBD OR "ulcerative colitis" OR Crohn* OR colitis OR "intestinal inflammation" OR "intestinal barrier" OR "intestinal mucosa*" OR "intestinal injury" OR "colorectal cancer" OR "colorectal carcinoma" OR "colon cancer" OR "rectal cancer" OR "colorectal tumor*" OR "colon tumor*" OR ("CRC" NEAR/5 (colorectal OR colon OR cancer OR tumor* OR carcinoma)) OR constipation OR "radiation-induced intestinal damage" OR "necrotizing enterocolitis") AND (therap* OR treat* OR treatment OR prevent* OR ameliorat* OR alleviat* OR immunotherap* OR "drug delivery" OR "anti-inflammatory" OR "anti inflammatory")) NOT TS=(diagnos* OR biosens* OR sensor* OR detector* OR imaging OR theranostic OR "breast cancer" OR melanoma OR glioma OR glioblastoma OR "lung cancer" OR "prostate cancer" OR "ovarian cancer" OR leukemia OR lymphoma OR "cervical cancer" OR diabetes OR obesity OR hyperuricemia OR gout OR "liver cancer" OR "hepatocellular carcinoma" OR "hepatic steatosis" OR "liver injury" OR "kidney disease" OR "renal disease" OR skin OR wound OR psoriasis OR Parkinson* OR Alzheimer*) | Publication years: 2015–2026; search date: July 3, 2026; document types: Article and Review; language: English | **488** |
| **Scopus** | Title, Abstract, and Keywords (TITLE-ABS-KEY) | TITLE-ABS-KEY((("engineered bacteri*" OR "genetically engineered bacteri*" OR "genetically modified bacteri*" OR "recombinant bacteri*" OR "engineered probiotic*" OR "genetically engineered probiotic*" OR "genetically modified probiotic*" OR "recombinant probiotic*" OR "synthetic probiotic*" OR "programmable probiotic*" OR "designer probiotic*" OR "smart probiotic*" OR "engineered commensal*" OR (("Escherichia coli Nissle" OR "E. coli Nissle" OR EcN OR "Lactococcus lactis" OR Lactobacillus OR Bifidobacterium OR Salmonella OR Bacteroides) W/5 (engineer* OR recombinant OR modified OR synthetic OR programmable OR express* OR secret*))) AND ("inflammatory bowel disease" OR IBD OR "ulcerative colitis" OR Crohn* OR colitis OR "intestinal inflammation" OR "intestinal barrier" OR "intestinal mucosa*" OR "intestinal injury" OR "colorectal cancer" OR "colorectal carcinoma" OR "colon cancer" OR "rectal cancer" OR "colorectal tumor*" OR "colon tumor*" OR ("CRC" W/5 (colorectal OR colon OR cancer OR tumor* OR carcinoma)) OR constipation OR "radiation-induced intestinal damage" OR "necrotizing enterocolitis") AND (therap* OR treat* OR treatment OR prevent* OR ameliorat* OR alleviat* OR immunotherap* OR "drug delivery" OR "anti-inflammatory" OR "anti inflammatory")) | Publication years: 2015–2026; search date: July 3, 2026; document types: Article and Review; language: English | **729** |

**Figure S1.** Author collaboration network in research on engineered bacteria and engineered probiotics for intestinal disease therapy.
